# Universality of Time–Temperature Scaling Observed by Neutron Spectroscopy on Bottlebrush Polymers

**DOI:** 10.1021/acs.nanolett.1c01379

**Published:** 2021-05-14

**Authors:** Karin J. Bichler, Bruno Jakobi, Victoria García Sakai, Alice Klapproth, Richard A. Mole, Gerald J. Schneider

**Affiliations:** † Department of Physics & Astronomy, Louisiana State University, Baton Rouge, Louisiana 70803, United States; ‡ Department of Chemistry, Louisiana State University, Baton Rouge, Louisiana 70803, United States; § ISIS Facility, Rutherford Appleton Laboratory, Harwell Science and Innovation Campus, Chilton, Didcot OX11 0QX, United Kingdom; ∥ Australian Nuclear Science and Technology Organisation, New Illawarra Road, Lucas Heights 2234, NSW, Australia

**Keywords:** Time−Temperature Superposition, Quasi-Elastic Neutron Scattering, Bottlebrush Polymer, Polydimethylsiloxane

## Abstract

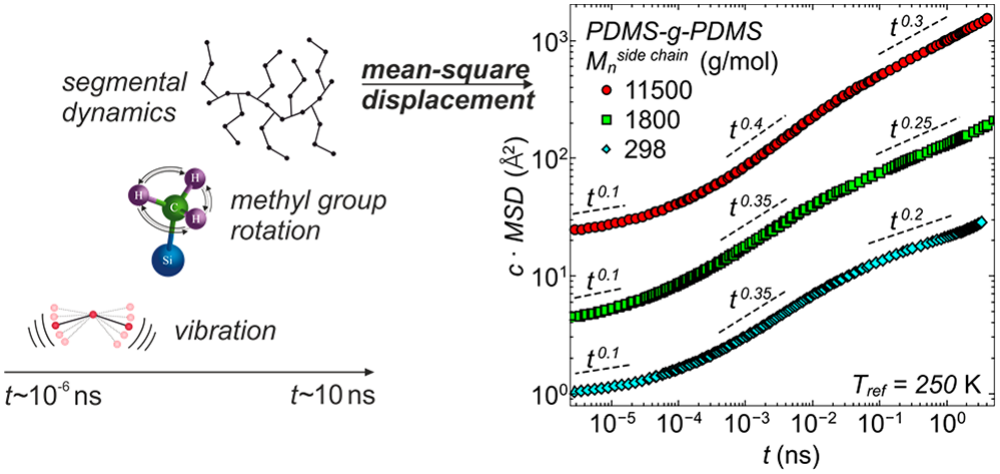

The understanding of materials requires access to the dynamics over many orders of magnitude in time; however, single analytical techniques are restricted in their respective time ranges. Assuming a functional relationship between time and temperature is one viable tool to overcome these limits. Despite its frequent usage, a breakdown of this assertion at the glass-transition temperature is common. Here, we take advantage of time- and length-scale information in neutron spectroscopy to show that the separation of different processes is the minimum requirement toward a more universal picture at, and even below, the glass transition for our systems. This is illustrated by constructing the full proton mean-square displacement for three bottlebrush polymers from femto- to nanoseconds, with simultaneous information on the partial contributions from segmental relaxation, methyl group rotation, and vibrations. The information can be used for a better analysis of results from numerous techniques and samples, improving the overall understanding of materials properties.

In polymer science and other material-specific disciplines, e.g., medicine^1^ or construction chemistry,^2−4^ dynamical processes occur over a wide time range.^5^ Because analytical instruments have finite time/frequency windows, only a fraction of the existing processes is accessible at a given temperature.^6−10^ A common approach to overcome this limitation is the empirical time–temperature superposition principle.^1,3,11,12^ In this technique, the analyzed quantity is measured at different temperatures and then shifted to one reference temperature, yielding a significantly extended time/frequency range. However, a breakdown of the time–temperature superposition principle seems to be very common, significantly impeding fundamental understanding of materials, especially polymer melts.

Samples are either classified as thermorheologically simple or complex.^13,14^ While in the first case the characteristic times scale with the temperature, heterogeneities and phase transitions, including the glass transition, are expected to cause a breakdown of thermorheological simplicity. Structural inhomogeneities seem to be a natural cause of this breakdown; however, dynamic heterogeneities may also have substantial impact. Both the influence on time–temperature scaling and the origin of the dynamic heterogeneities are still under debate and may be linked to structural heterogeneities, different chemical surroundings, and could also be caused by a superposition of different relaxation processes.^15,16^ Below we report the apparent breakdown of the universal time–temperature scaling approach and illustrate a more generic principle, which specifies the minimum requirement to compare experimental results from different techniques, related to contributions of different relaxation processes.

We take advantage of length- and time-scale information in quasi-elastic neutron scattering (QENS) experiments to examine the influence of superpositioned processes on the violation of the time–temperature superposition principle. The process focuses on the glassy dynamics or -process captured at small length scales corresponding to dimensions smaller than the size of Gaussian blobs.^5^ In the related momentum transfer range, , the dynamic correlation function probes the heterogeneous relaxation associated with the segmental relaxation. Thus, the selection of the length-scale range excludes contributions of the homogeneous large-scale polymer dynamics typically associated with Rouse relaxation and also the transition region between Rouse and the more local segmental relaxation, facilitating focus on the various local heterogeneities, .

The dynamical heterogeneities of two different origins are introduced to study the influence on the time–temperature superposition via the mean-square displacement (MSD) obtained from quasi-elastic neutron scattering (QENS). The systems of study are poly(dimethylsiloxane)-based homopolymer bottlebrushes, PDMS-*g*-PDMS, with varying side chain and similar backbone molecular weights of  g/mol and  g/mol, synthesized by anionic polymerization and investigated by dielectric spectroscopy and QENS.^17,18^ Despite being a structurally very complex polymer, there seems to be no indication of any thermorheologically complex behavior.^17^ As illustrated below, this unexpected observation is a consequence of the experimental technique’s specific sensitivity and reflects a different superposition that is method specific of the underlying molecular processes. The presented analysis is exemplified using one bottlebrush polymer,  g/mol, followed by a comparison of all three samples.

The first dynamical heterogeneity is a consequence of the three different relaxation processes in PDMS, visible at  Å^–1^.^19^ At high temperatures, the dynamics accessible for PDMS-based polymers in the QENS time window are governed by segmental relaxation, whereas at lower temperatures, rotational jump motions of the methyl groups are captured, as shown in Figure 1a.^18^ Additional fast motions, like vibrations, can be well separated, but give a noticeable contribution to the mean-square displacement. Changing the temperature shifts the dynamical processes through the accessible time window and allows to either capture the pure relaxation phenomena or a mixture of them. Measurements in this study are carried out at temperatures at which our experimental data reflect only individual processes because of separated processes. Knowing the individual processes and their characteristic behavior provides the opportunity to calculate experimental data in the intermediate temperature range in which more than one process contributes.

**Figure 1:**
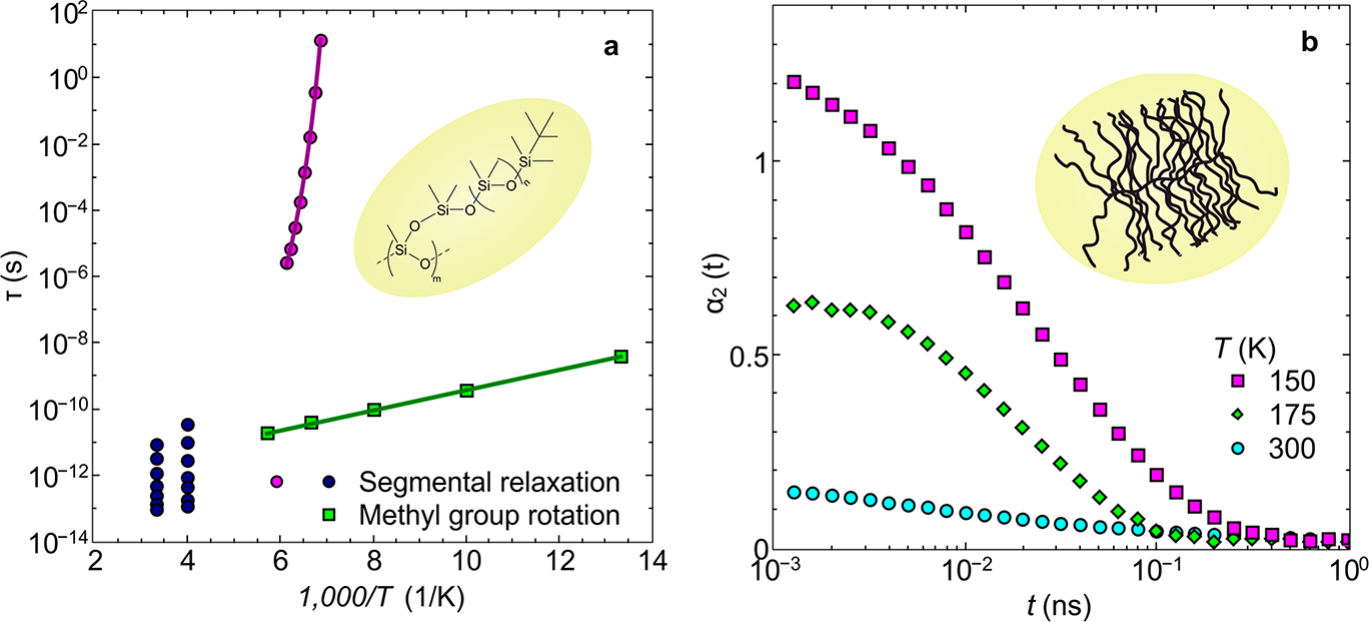
Dynamical heterogeneity of the example PDMS-*g*-PDMS bottlebrush polymer with  g/mol. (a) Relaxation times, , vs  for the different dynamical processes occurring in PDMS. The solid purple line is the best description with the Vogel–Fulcher–Tammann equation for the segmental relaxation times, obtained by dielectric spectroscopy.^17^ The solid green line is the best description with the Arrhenius equation for the relaxation times of the methyl group rotations, obtained by QENS.^18^ The solid blue symbols are the segmental relaxation times for different momentum transfers,  Å^–1^ from top to bottom, obtained by QENS.^18^ Inset: Chemical structure of PDMS-*g*-PDMS. (b) Non-Gaussian parameter, , vs time, , for the PDMS-*g*-PDMS bottlebrush polymer for three selected temperatures,  K. Inset: Pictorial representation of the morphology of PDMS-*g*-PDMS.

The second dynamical heterogeneity in our sample is associated with the intrinsic heterogeneity of the segmental relaxation and is magnified because of the branched nature of the bottlebrush architecture.^20^ The grafting of side chains onto a polymer backbone results in a broader distribution of relaxation times.^17^

Dynamic heterogeneity is specified by the non-Gaussian parameter, , which is theoretically defined as . It is represented by the ratio of the fourth moment, , and the second moment squared, , of a distribution function. Distinct nonzero values of  indicate deviations from a Gaussian distribution of relaxation times, i.e., deviations from homogeneous dynamics.^16^ As seen in Figure 1b,  displays values well above zero. For all temperatures it shows a time dependence, indicating an increase in homogeneity at longer times.^21^

We use the mean-square displacement representation of the time–temperature scaling of the incoherent intermediate scattering function, , resulting from the combination of three different QENS instruments capturing three orders of magnitude in the time scale, ranging from pico- to nanoseconds.^18^
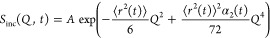
1

The different temperature data sets are converted into the mean-square displacement, , of all protons in the system by using the cumulant series expansion which has been shown to produce highly reliable information by plotting data in the Guinier representation, i.e.,  vs  (Figure S1).^15,16,22−24^

This model-independent analysis allows the identification of distinct processes apparently separated in temperature. At high temperatures the segmental relaxation, at intermediate-to-low temperatures the methyl group rotations, and at low temperatures the Debye–Waller factor contribute to the mean-square displacement and the resulting power laws (Figure 2a). The mean-square displacement of the methyl group rotation is found to show a pronounced plateau expected for the constrained nature of the motion, whereas the segmental dynamics show a continuous increase.^25,26^

**Figure 2:**
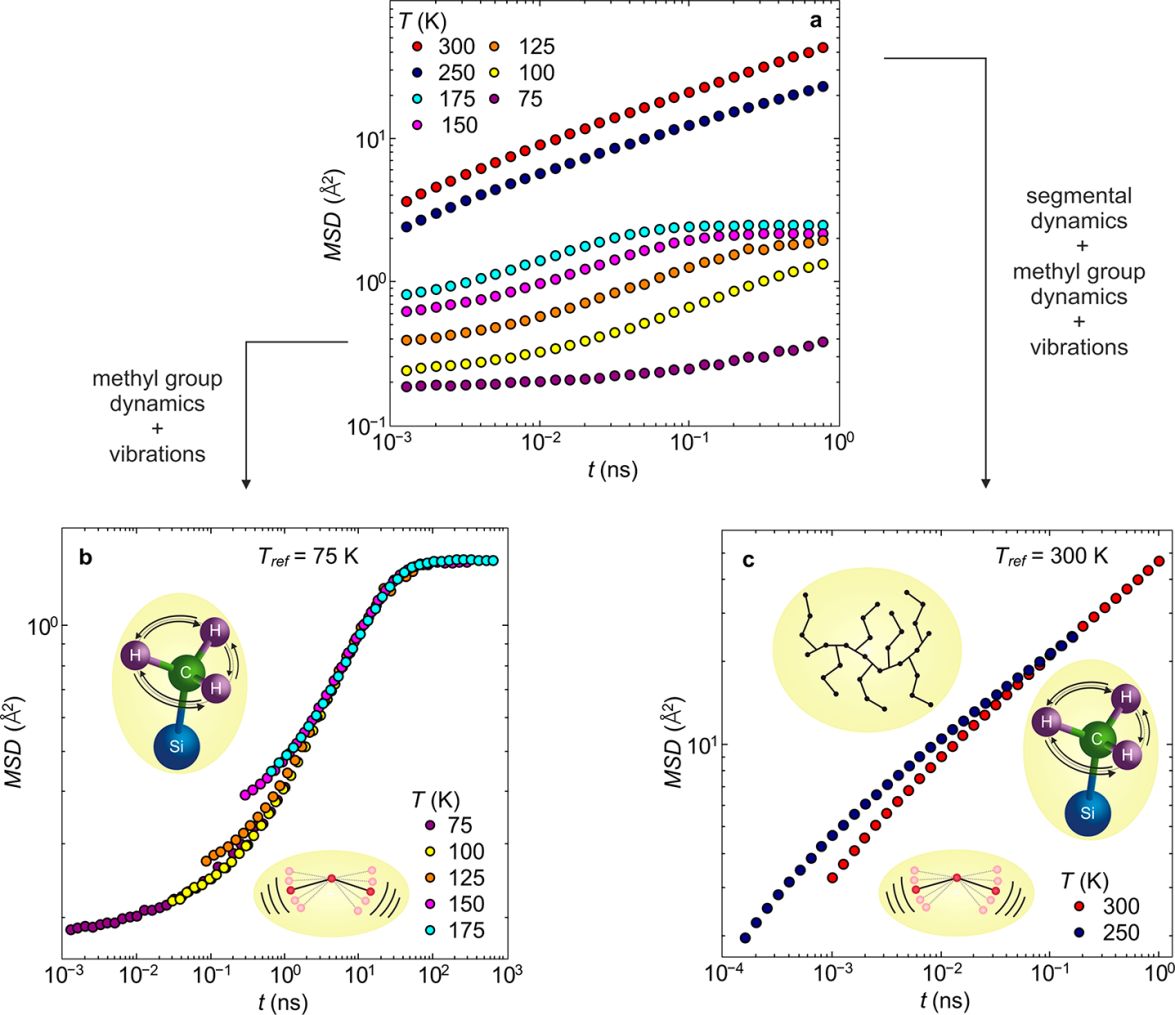
Mean-square displacement of the example PDMS-*g*-PDMS with  g/mol, obtained from QENS. (a) Mean-square displacement, , vs time, , as obtained from the intermediate scattering function, , by using the cumulant series expansion, eq 1. (b) Mean-square displacement, , vs time, , for the low temperatures, showing contributions from methyl group rotations and fast vibrations at the reference temperature,  K, not fulfilling the time–temperature superposition principle. Insets: Pictorial representation of vibrations and methyl group rotations. (c) Mean-square displacement, , vs time, , for the high temperatures, showing contributions from segmental dynamics, methyl group rotation, and fast vibrations at the reference temperature,  K, not fulfilling the time–temperature superposition principle. Inset: Pictorial representation of vibrations, methyl group rotations, and segmental dynamics.

The set of mean-square displacement graphs show strong similarities, thus hinting at the possibility of a valid time–temperature scaling. Yet, a test of the classic time–temperature superposition principle by introducing shift factors in horizontal and vertical directions reveals strong disparities (Figure 2b for methyl group dynamics and Figure 2c for segmental dynamics). In particular, the breakdown of the time–temperature superposition at high temperatures is striking, though one would expect that it should work best at high temperatures. A closer look at both failed attempts of constructing a master curve shows that the discrepancies are most pronounced on short scales and in the region of relatively small displacements. At longer times and relatively high displacements, in contrast, a good overlap is reached.

Considering the possible sources of heterogeneity (Figure 1), the coexistence of two or more processes in the momentum transfer region for which the MSD has been calculated could already be responsible for at least a part of the mismatch of our data illustrated in Figure 2b and Figure 2c. Therefore, consider the influence of the coexistence of two or more processes by taking advantage of the strongest individual contribution on different time scales, i.e., vibrations at short times, methyl group rotations at intermediate times, and segmental relaxation at long times. Hence, using the occurrence of the processes on very different time scales and the additivity of the mean-square displacement allows for the examination of how much of the mismatch can be related to the existence of multiple processes in polymers. This essentially reflects the time–temperature superposition principle at the single molecular process level and identifies the individual contributions as the major source of the breakdown of the time–temperature scaling applied to the directly measured data.

The vibrations governing short times are typically considered via the atomistic mean-square displacement, , included in the Debye–Waller factor,  (Figure S2a).^5,27^ Access to  is provided by the description of the intermediate scattering function, .^18^ The atomistic mean-square displacement has a pronounced temperature dependence but is time independent within our time and -window, giving a constant contribution to the respective mean-square displacement as shown in Figure S2b. The values of  are comparatively small (Figure S2), thus subtraction in the temperature range of  K results in the pure methyl group motion. Applying the Arrhenius type temperature scaling for methyl group rotation (Figure S3) leads to temperature superposition as illustrated in Figure 3a. Within the experimental accuracy, excellent agreement is found up to  K, i.e., for the entire temperature range for which the motion is visible. Thus, time–temperature scaling seems to be applicable to methyl group rotation.

**Figure 3:**
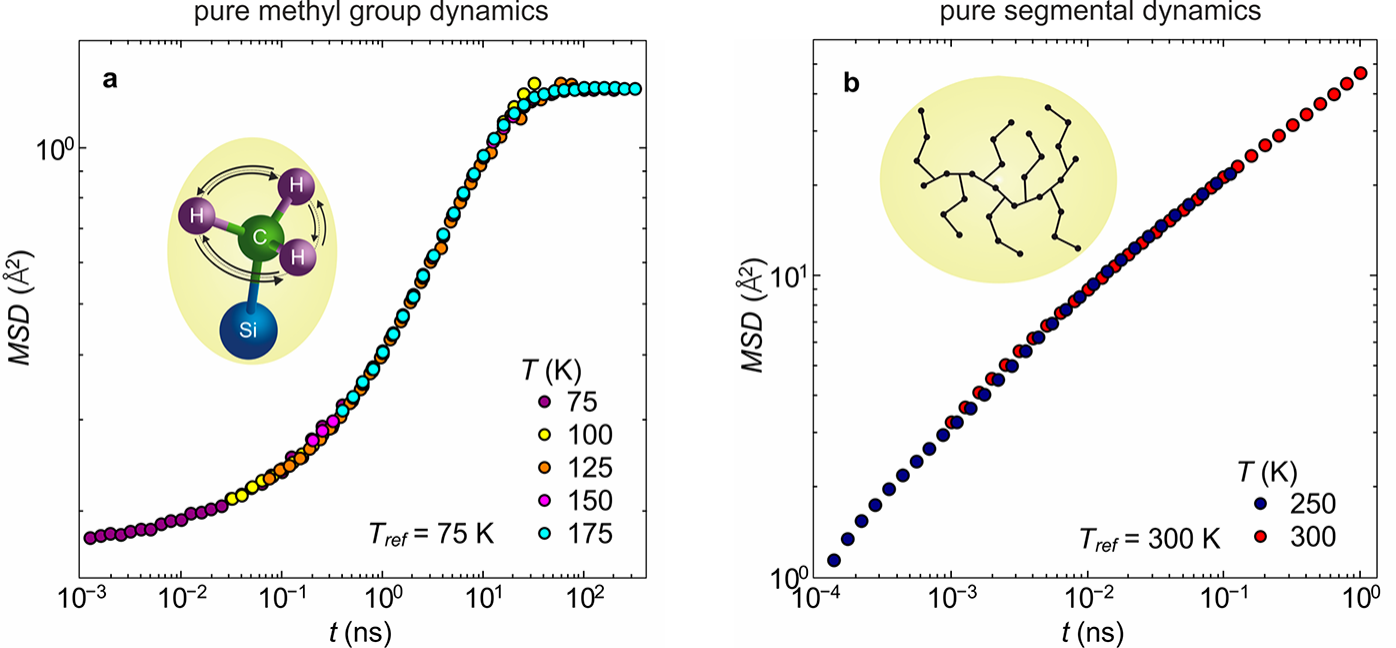
Partial mean-square displacements relating to single processes, specifically methyl group rotation and segmental dynamics for the example PDMS-*g*-PDMS bottlebrush polymer with  g/mol. Partial mean-square displacement, , vs time, of (a) the methyl group rotation at the reference temperature,  K, and (b) of the segmental dynamics at  K. Insets: Pictorial representations of the methyl group rotation and segmental dynamics, respectively. With the partial mean-square displacements in hand, the temperature-dependent full mean-square displacement can be reconstructed.

At higher temperatures, the mean-square displacement of the segmental relaxation contains contributions from all three motions, including vibrations and methyl group rotations. A shifting of the master curve of the mean-square displacement of the pure methyl group dynamics by extrapolating the gained shift factors to the data at the two high temperatures ( K and  K) allows the calculation of the mean-square displacement of the segmental dynamics alone, resulting in the single mean-square displacement of the pure segmental dynamics.

Similar to the methyl group rotation, the segmental mean-square displacement can also be combined into a single master curve avoiding artificial scaling on the time axis by using shift factors obtained by dielectric spectroscopy. The superpositioned data point to a well-working time–temperature superposition for the segmental relaxation. Analysis of the QENS data in this way shows that in fact the time–temperature superposition principle is valid over the whole time and temperature range, including at temperatures well below the glass-transition temperature, if the data are carefully analyzed to reflect only one single dynamical process, even if heterogeneous processes are studied. Therefore, this method enables to increase the experimental window from three orders of magnitude up to five and four for the methyl group and segmental relaxation, respectively, on a single process level.


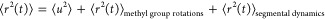
2In the full mean-square displacement, the time scale is expanded to seven orders of magnitude as shown in Figure 4. A self-consistent verification for the approach used is achieved by comparing the created full mean-square displacement with the original mean-square displacement data of the reference temperature,  K. In the restricted time range of the original mean-square displacement ( ps  ns) from Figure 2a, both data sets, i.e., the constructed full mean-square displacement and the original mean-square displacement at  K, agree very well (Figure S5).

**Figure 4:**
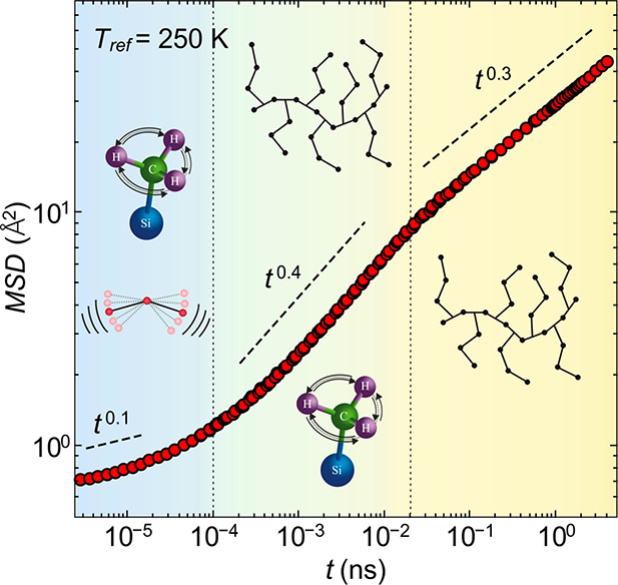
Full proton mean-square displacement of the example PDMS-*g*-PDMS bottlebrush polymer with  g/mol. Mean-square displacement, , vs time, , for all contributing motions, segmental dynamics, methyl group rotations, and fast vibrations, obtained by the combination of the single processes at the reference temperature,  K. Dashed lines indicate the resulting power laws for the respective time scale. Insets: Pictorial representation of vibrations, methyl group rotations, and segmental dynamics.

This result has a remarkable consequence, as it reflects the strong influence of the individual process contributions to the full mean-square displacement. The absence of strong deviations points to a lesser importance of the heterogeneities of the individual processes to the breakdown of the known time–temperature superposition principle. Thus, the importance of the individual contributions of different processes explains why the validity of time–temperature superposition seems to have different temperature ranges when it comes to different techniques. For example, dielectric spectroscopy does not detect the methyl group rotation, and therefore, the frequency scaling of the segmental relaxation can be observed at least down to temperatures . Though dielectric spectroscopy has an extremely broad frequency window, below this temperature the experimental signal leaves the finite frequency range.^17^ Hence, lower temperatures have not been accessed. The current QENS results suggest that the observation continues to much lower temperatures.

The last point to discuss in more detail is the mean-square displacement. Based on the separate constituting processes, three regions can be identified (Figure 4), each dominated by different relaxations. At short times, a mixture of vibrations and methyl group rotations cause a power law of . This is followed by  in the intermediate time scale, which is seen as an interplay of methyl group rotations and segmental relaxation. In the long-time limit the segmental relaxation dominates the mean-square displacement, showing a pronounced power law of .

Using the same ansatz for the other two samples, i.e., PDMS-*g*-PDMS with  g/mol and  g/mol, results in the full proton mean-square displacement of the PDMS-*g*-PDMS bottlebrush polymer having elongated shapes^17^ (Figure 5). While the three different dynamical regions are visible in all three samples, the power law dependence of the mean-square displacement changes depending on the side chain length. The short time region, vibrational motions and methyl group rotations, shows the same power law of  independent of the side chain length. Continuing to the intermediate time region, the interplay of methyl group rotations and segmental relaxation, an increasing power law from  to  is observable. The same effect is visible for the long-time region, where the dynamics are dominated by the segmental relaxation. Here, the power law increases from  to  with increasing the side chain length. This allows the assumption that with increasing molecular weight of the side chains, i.e., with increasing distance from the backbone, the segmental dynamics is less confined, resulting in steeper power laws, eventually approaching the behavior of linear polymers at sufficiently long side chain lengths.

**Figure 5:**
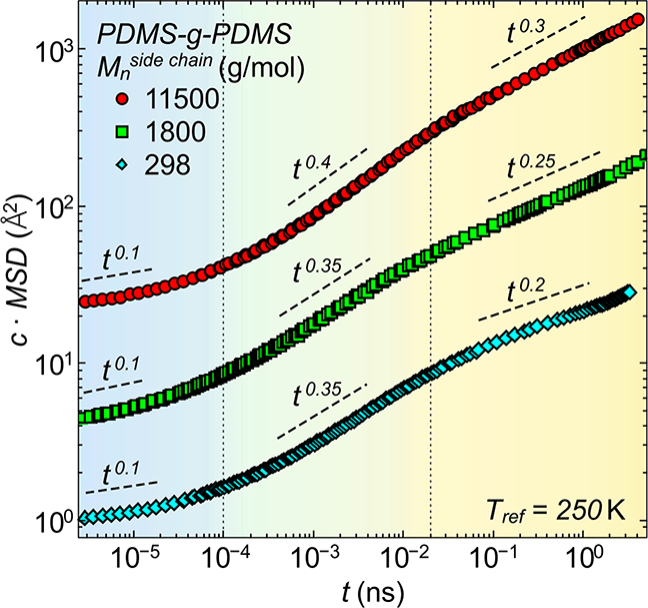
Full proton mean-square displacement of the three different PDMS-*g*-PDMS bottlebrush polymers. Mean-square displacement, , vs time, , for all contributing motions, segmental dynamics, methyl group rotations, and fast vibrations, obtained by the combination of the single processes at the reference temperature,  K. Dashed lines indicate the resulting power laws for the respective time scale. The MSD for the  and  are shifted vertically by factors of  and , respectively, for clarity.

However, these results deviate from power laws expected for polymers, including Rouse dynamics and reptation.^5,28^ The previous paragraph explains that a part of these deviations comes from the experimental observation of the entire proton motion reflecting multiple processes as mirrored by the full mean-square displacement, while typical theoretical equations usually pick a certain process such as segmental relaxation. However, the closer inspection of the partial mean-square displacements, Figure 3, shows a behavior that does not reflect the relaxation of an ideal polymer chain, which may be attributed to the bottlebrush polymer itself. To date, there is no theory available that would explain the mean-square displacement of this branched polymer with complex architecture.

In conclusion, the approach presented here shows how the time–temperature superposition principle can successfully be used for dynamically heterogeneous samples, as long as the dynamics can be split into single processes based on their respective time and temperature dependences. It is not limited to a bottlebrush shape; it could also be applied to any material as long as the length and time scales can be separated. This enables a significant extension of the available time/frequency range, typically limited for experimental methods commonly used to study polymer dynamics, e.g., QENS, rheology, or NMR, etc. and for a multitude of possible samples. We anticipate that this analysis will have a notable impact on the data analysis and understanding of resulting master curves, enhancing the data interpretation based on a single-process approach.

## Supporting Information Available

Description of the used method, supporting analysis steps for the example PDMS-*g*-PDMS with  g/mol, supporting data for PDMS-*g*-PDMS bottlebrush polymers with  g/mol, and supporting data for PDMS-*g*-PDMS bottlebrush polymers with  g/mol (PDF)
