# Supplementary material for: Universality of Time–Temperature Scaling Observed by Neutron Spectroscopy on Bottlebrush Polymers
Source: Nano Lett. 2021 May 14;21(10):4494–9. doi: 10.1021/acs.nanolett.1c01379 (PMC8289293; doi:10.1021/acs.nanolett.1c01379)
Supplement: Supplementary file 1 — nl1c01379_si_001.pdf [file nl1c01379_si_001.pdf]

Supporting Information

Universality of Time-Temperature Scaling Observed by Neutron Spectroscopy on Bottlebrush Polymers

Authors: Karin J. Bichler,[1†*] Bruno Jakobi,[2] Victoria García Sakai,[3] Alice Klapproth,[4] Richard A. Mole,[4] Gerald J. Schneider[1,2*]

Affiliations:

[1]Department of Physics & Astronomy, Louisiana State University, Baton Rouge, Louisiana 70803, United States.

[2]Department of Chemistry, Louisiana State University, Baton Rouge, Louisiana 70803, United States.

[3]ISIS Facility, Rutherford Appleton Laboratory, Harwell Science and Innovation Campus, Chilton Didcot, OX11 0QX, United Kingdom

[4]Australian Nuclear Science and Technology Organisation, New Illawarra Road, Lucas Heights 2234, NSW, Australia

[†]Present address: Department of Chemistry, Louisiana State University, Baton Rouge, Louisiana 70803, United States.

*Correspondence to: kbichler@lsu.edu, gjschneider@lsu.edu

Method

Samples

The samples used for quasi-elastic neutron scattering (QENS) experiments, were PDMS-g-PDMS bottlebrush polymer with varying side chain length, $M_n^{side\ chain} = 298, 1800, 11500$ g/mol, and similar backbone length, $M_n^{backbone} = 16500, 13500$ g/mol, synthesized based on anionic ring opening polymerization and characterized by Gel Permeation Chromatography (GPC), and Nuclear Magnetic Resonance (NMR). A more detailed description of the synthesis can be found in Jakobi *et al.*[S1]

Neutron Scattering Experiments

Dynamical studies, based on the intermediate scattering function, $S_{inc}(Q,t)$, on these samples have been performed by QENS and have been published in Bichler *et al.*[S5] Hereby, a combination of three different spectrometers has been used to capture a time range of three orders of magnitude, i.e., $t = 1$ ps to $t = 1$ ns. The short times were covered by the time-of-flight spectrometer Pelican[S2] and the long times by the backscattering spectrometer EMU[S3], both located at ANSTO, Sydney, Australia. For the intermediate times, the time-of-flight backscattering instrument IRIS[S4], at the spallation source ISIS, Didcot, UK was used. Transforming the data into the time domain, results in the incoherent intermediate scattering function, $S_{inc}(Q,t)$. This allows a combination of the data of the three instruments and subsequently enables the combined analysis of $S_{inc}(Q,t)$ based on the different relaxation processes.[S5] The time dependence of $S_{inc}(Q,t)$ for all temperatures measured and for all three samples can be found in Bichler *et al.*[S5]

Supporting Analysis Steps for the example PDMS-g-PDMS with $\mathbf{M_n^{side\ chain}} = \mathbf{11500}$ g/mol

Mean-Square Displacement Analysis

The mean-square displacement (MSD) analysis is based on the cumulative serial expansion of the incoherent intermediate scattering function, $S_{inc}(Q,t)$, following equation (1) in the main text. Hereby, $\ln\left(S_{inc}(Q,t)\right)$ is represented as a function of Q^2 for selected time values, t. Describing this representation with equation (1) leads to two fit parameters, the non-Gaussian parameter, $\alpha_2(t)$, and the mean-square displacement, MSD ($\langle r^2(t)\rangle$), both depending on time, t. This analysis way is illustrated in Fig. S1 exemplarily for 8 different time values. For the main text, this analysis has been done for 31 different times values, resulting in 31 values for $\alpha_2(t)$ and for the mean-square displacement, MSD. Both are illustrated in Fig. 1b and Fig. 2a, of the main text, respectively.

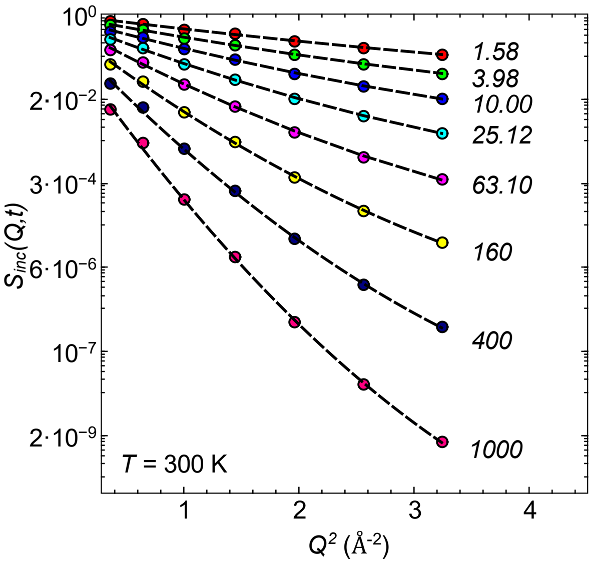

Fig. S1. Analysis of the experimental intermediate scattering function, $S_{inc}(Q,t)$, with the cumulant series expansion. Intermediate scattering function, $S_{inc}(Q,t)$, represented in the common logarithm vs. Q^2 for eight different time values in picoseconds, as indicated, at the temperature $T = 300$ K. Dashed lines are the description with equation (1) from the main text.

Adjustment for Fast Vibrations

In order to adjust the mean-square displacement of the methyl group and the segmental dynamics for fast vibrations, the atomistic mean-square displacement has been used. It is included in the Debye-Waller Factor and obtained from the incoherent intermediate scattering function analysis. These values have been subtracted from the respective temperatures of the original mean-square displacement data (Fig. 2a) to gain the single process mean-square displacement. The resulting temperature dependence of the atomistic mean-square displacement is shown in Fig. S2 including a dashed line, which serves as a guide for the eye.

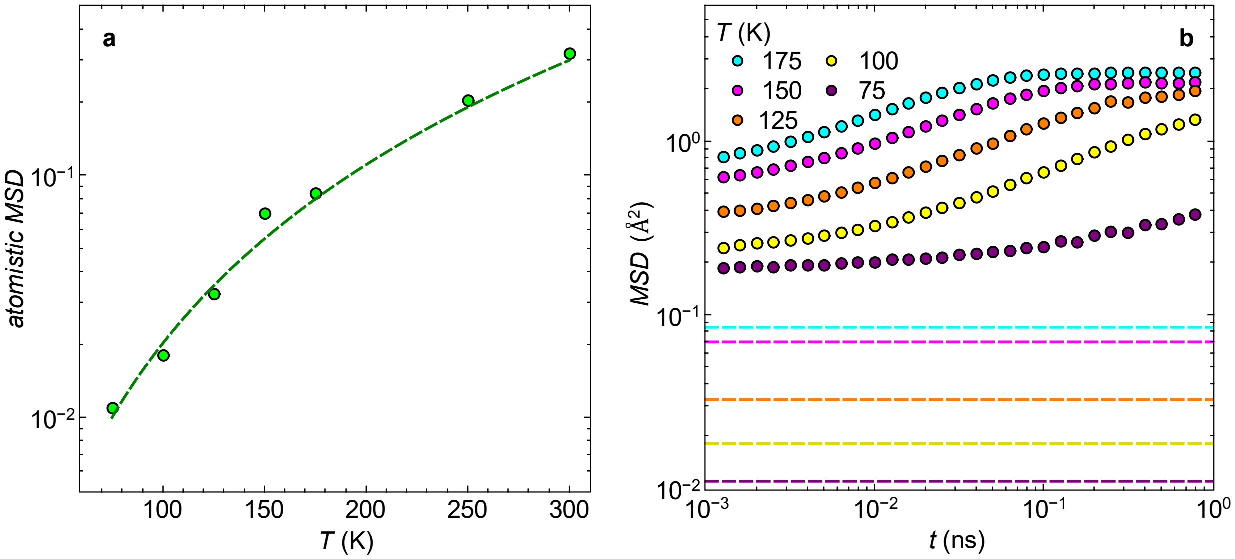

Fig. S2. Atomistic mean-square displacement of the PDMS-g-PDMS with $M_n^{side\ chain} = 11500$ g/mol. (a) Atomistic mean-square displacement, MSD, vs. temperature, T, for the PDMS-g-PDMS bottlebrush polymer. Dashed line is a guide for the eye. **(b)** Mean square displacement, MSD, vs. time, t, for the methyl group rotations. Dashed lines represent the time-independent atomistic mean square displacement at the respective temperatures as indicated.

Shift Parameter

To create the mean-square displacement of the single processes, time, a_T, and displacement, b_T, scaling has been applied. Hereby, for the methyl group mean-square displacement shift factors for both directions have been used, while for the segmental mean-square displacement only the time axis has been scaled. In case of the methyl group, time shift factor, a_T, and relaxation time, τ_ℓ, show a similar temperature scaling, which points to a time-temperature superposition principle (purple squares in Fig. S3a). The temperature dependence can be described with an Arrhenius law, with the activation energy, E_A, being the same as obtained for the temperature dependence of the relaxation times, τ_ℓ.

$$a_T = a_0 \cdot \exp\left(\frac{E_A}{RT}\right) \tag{S1}$$

The shift factors, b_T, show a linear relationship with temperature as illustrated in Fig. S3b, which is described by

$$b_T = m \cdot T + b_0 \tag{S2}$$

The associated fit parameter, describing the temperature dependence for both shift factors are summarized in Table S1.

The mean-square displacement for the segmental dynamics needs only to be shifted along the time axis. The shift parameters used follow the Vogel-Fulcher-Tammann (VFT) behavior known from dielectric spectroscopy experiments on this sample.[S1]

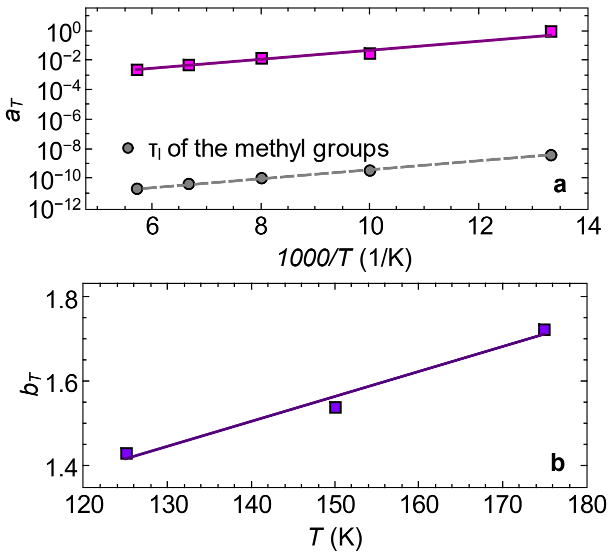

Fig. S3. Temperature dependence of the obtained shift parameters for the methyl group mean-square displacement. (a) Shift parameter, a_T, vs. $1000/T$. Solid purple line is the best description with the Arrhenius law, equation (S1). Grey symbols and dashed grey lines are the temperature dependence of the relaxation times, τ_ℓ, obtained for the methyl group rotation and included for comparison. **(b)** Shift parameter, b_T, vs. temperature, T. Solid line is the best description with the linear relationship and parameters defined in equation (S2).

Table S1. Fit parameters for the temperature dependencies. Temperature dependence of the shift factors, a_T and b_T as described by equations (S1), and (S2), respectively. Errors are the standard deviations of the respective quantity.

	a_0	E_A (kJ/mol)
a_T	$(4.2 \pm 0.6) \cdot 10^{-5}$	(5.8 ± 0.4)

	m (1/K)	b_0
b_T	(0.006 ± 0.001)	(0.7 ± 0.1)

Parametrization of the Pure Segmental Mean-Square Displacement

To simplify the summation of the pure methyl group mean-square displacement and the pure segmental mean-square displacement, the latter one was interpolated by the mathematical expression

$$\langle r^2(t)\rangle = at^b + a_1 t^{b_1} + a_2 t^{b_2} \tag{S3}$$

with $a = 502$, $b = 0.6$, $a_1 = -800$, $b_1 = 0.7$, $a_2 = 324$, and $b_2 = 0.74$ as illustrated in Fig. S4.

The aim was to find a parametrization of the segmental mean-square displacement for further data treatment, allowing to create the master curve of the segmental mean-square displacement in the same time range with exactly the same points as obtained for the methyl group mean-square displacement master curve. This allows easier pointwise addition of the partial mean-square displacements, resulting in the overall mean-square displacement (Fig. 4b).

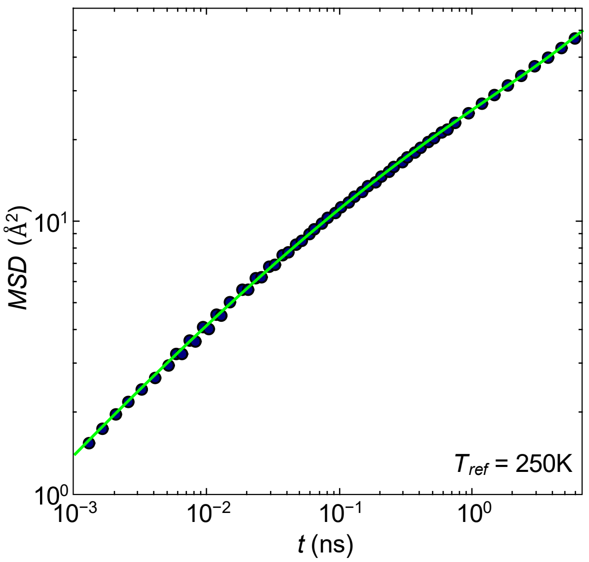

Fig. S4. Parametrization of the pure segmental dynamics mean-square displacement. Mean-square displacement, MSD, vs. time, t, at the reference temperature $T_{ref} = 250$ K. Solid line is the parametrization with equation (S3).

Experimental verification of the approach used for creating the final mean-square displacement.

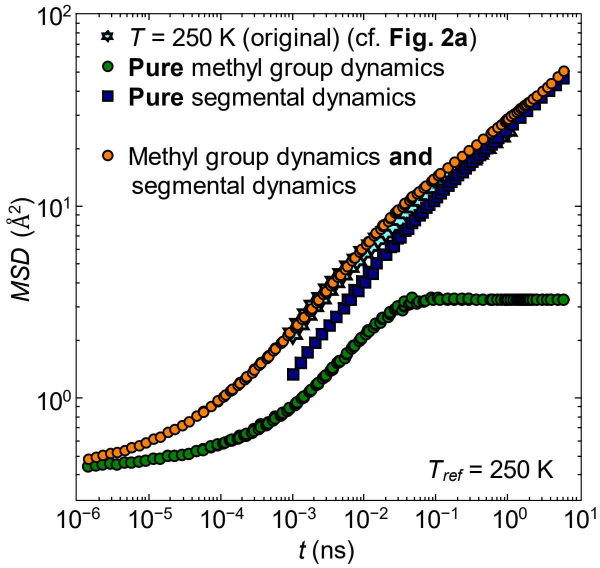

Fig. S5. Experimental verification of the approach used for creating the final mean-square displacement. Mean-square displacement, MSD, vs. time, t, for the single methyl group rotation (solid green circles), for the single segmental dynamics (solid blue squares), for the original mean-square displacement, as obtained from the intermediate scattering function (solid cyan stars) and the combined mean-square displacement of methyl group and segmental dynamics (solid orange circles) without the atomistic mean-square displacement.

Supporting Data for PDMS-g-PDMS bottlebrush polymers with $M_n^{side\ chain} = 1800$ g/mol

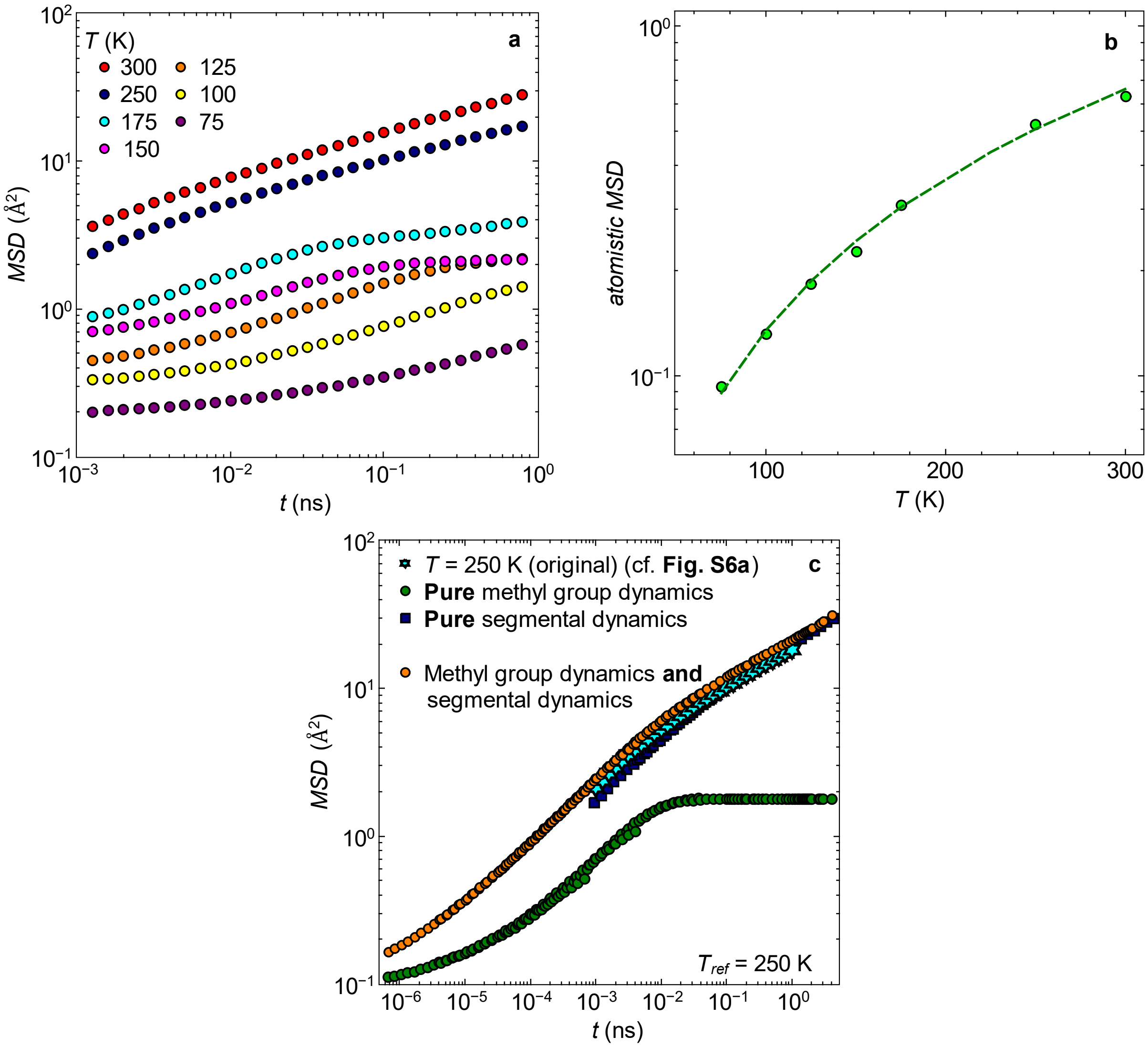

Fig. S6. Supporting data for the PDMS-g-PDMS bottlebrush polymer with $M_n^{side\ chain} =$ 1800 g/mol. (a) Mean-square displacement, MSD, vs. time, t, as obtained from the intermediate scattering function, $S(Q,t)$, by using the cumulant series expansion, equation (1) from the main text. **(b)** Atomistic mean-square displacement, MSD, vs. temperature, T. Dashed line is a guide for the eye. **(c)** Mean-square displacement, MSD, vs. time, t, for the single methyl group rotation (solid green circles), for the single segmental dynamics (solid blue squares), for the original mean-square displacement, as obtained from the intermediate scattering function (solid cyan stars) and the combined mean-square displacement of methyl group and segmental dynamics (solid orange circles) without the atomistic mean-square displacement.

Supporting Data for PDMS-g-PDMS bottlebrush polymers with $M_n^{side\ chain} = 293$ g/mol

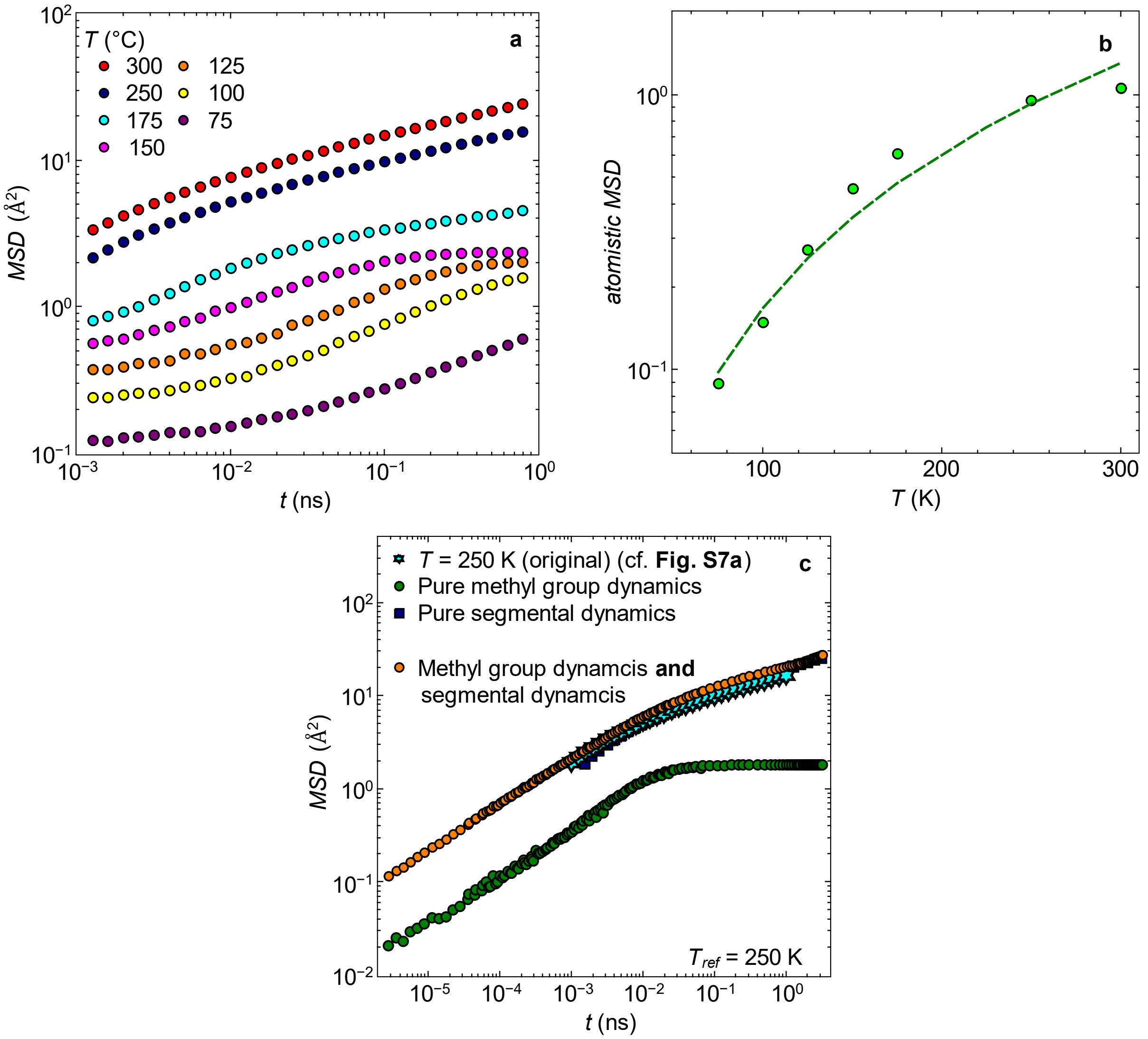

Fig. S7. Supporting data for the PDMS-g-PDMS bottlebrush polymer with $M_n^{side\ chain} =$ 293 g/mol. (a) Mean-square displacement, MSD, vs. time, t, as obtained from the intermediate scattering function, $S(Q,t)$, by using the cumulant series expansion, equation (1) from the main text. **(b)** Atomistic mean-square displacement, MSD, vs. temperature, T. Dashed line is a guide for the eye. **(c)** Mean-square displacement, MSD, vs. time, t, for the single methyl group rotation (solid green circles), for the single segmental dynamics (solid blue squares), for the original mean-square displacement, as obtained from the intermediate scattering function (solid cyan stars) and the combined mean-square displacement of methyl group and segmental dynamics (solid orange circles) without the atomistic mean-square displacement.

Additional Figures on the example of PDMS-g-PDMS with $M_n^{side\ chain} = 11500$ g/mol

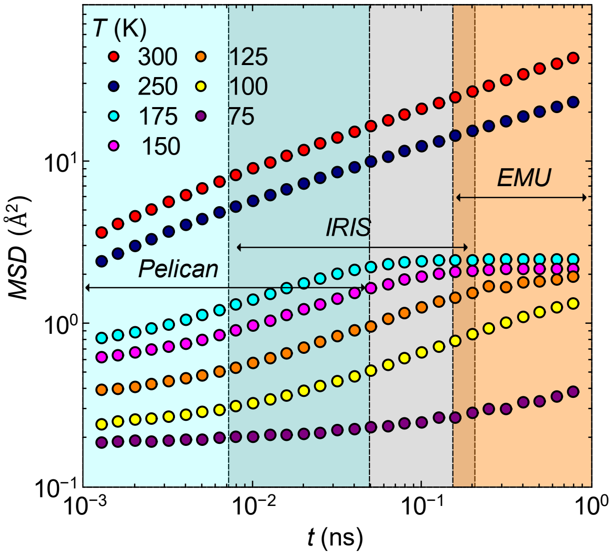

Fig. S8: Illustration of the time scales of the three different neutron spectrometers. Mean-square displacement, MSD, vs. time, t, as obtained from the intermediate scattering function, $S(Q,t)$, by using the cumulant series expansion, equation (1). Shaded areas indicate the time scales of the different spectrometer. Short times: Pelican, intermediate times: IRIS, and long times: EMU.

References:

S1. Jakobi, B.; Bichler, K. J.; Sokolova, A.; Schneider, G. J., Dynamics of PDMS-g-PDMS Bottlebrush Polymers by Broadband Dielectric Spectroscopy. *Macromolecules* **2020,** *53* (19), 8450-8458.

S2. Yu, D.; Mole, R.; Noakes, T.; Kennedy, S.; Robinson, R., Pelican — a Time of Flight cold Neutron Polarization Analysis Spectrometer at OPAL. *Journal of the Physical Society of Japan* **2013,** *82* (Suppl.A), SA027.

S3. de Souza, N. R.; Klapproth, A.; Iles, G. N., Emu: High-Resolution Backscattering Spectrometer at ANSTO. *Neutron News* **2016,** *27* (2), 20-21.

S4. Demmel, F.; McPhail, D.; French, C.; Maxwell, D.; Harrison, S.; Boxall, J.; Rhodes, N.; Mukhopadhyay, S.; Silverwood, I.; Sakai, V. G.; Fernandez-Alonso, F., Tof-Backscattering Spectroscopy at the ISIS Facility: Status and Perspectives. *Journal of Physics: Conference Series* **2018,** *1021*, 012027.

S5. Bichler, K. J.; Jakobi, B.; Sakai, V. G.; Klapproth, A.; Mole, R. A.; Schneider, G. J., Short-Time Dynamics of PDMS-g-PDMS Bottlebrush Polymer Melts Investigated by Quasi-Elastic Neutron Scattering. *Macromolecules* **2020,** *53* (21), 9553-9562.
